# Disseminated Gonorrhea

**DOI:** 10.5811/cpcem.2019.9.44272

**Published:** 2019-11-19

**Authors:** Janelle Estrada, Shane Sergent, John Ashurst

**Affiliations:** Kingman Regional Medical Center, Department of Emergency Medicine, Kingman, Arizona

## Abstract

Sexually transmitted infections have risen sharply over the last decade in the United States. The incidence of gonorrhea has risen to 172 reported cases per 100,000 people over the past year. This likely represents an under-representation due to many cases going unreported. Disseminated gonorrhea can present with nonspecific symptoms including arthralgia, cutaneous lesions, or tenosynovitis. Diagnosis is based upon a degree of high clinical suspicion and serology. Emergency department treatment includes ceftriaxone and azithromycin.

## CASE PRESENTATION

A 23-year-old male presented to the emergency department (ED) due to a three-day history of a painful, swollen, and erythematous left third digit. He also noted that over the preceding day a black blister had formed on the affected digit. He admitted to a history of intravenous drug use within the prior week but not within the affected digit. He also admitted to unprotected sexual intercourse with multiple partners. He also noted penile discharge one week prior with associated dysuria. Physical examination revealed an erythematous and swollen left third digit with a hemorrhagic bullae (Image). Urine polymerase chain reaction was positive for gonorrhea. The patient was treated with ceftriaxone and azithromycin and admitted for further care. Blood cultures and a wound culture were negative at five days.

## DIAGNOSIS

The incidence of sexually transmitted infections in the United States has been on the rise over the last decade. Since 2009, reported cases of gonorrhea have increased by 75.2% and by 18.6% in the last two years.^1^ In 2017 alone, a total of 555,608 new cases of gonorrhea were reported to the Centers for Disease Control and Prevention.^1^ Although cases of gonorrhea are relatively common for the emergency physician, disseminated gonorrhea occurs in 0.2–1.9% of all patients infected with *Neisseria (N.) gonorrhoeae.*^2^ Cutaneous manifestations are generally nonspecific but can present as herpetiform or non-herpetiform pustules, necrotic vesicles, bullae, or tender purpuric papules.^2^ Diagnosis is confirmed by either identification of *N. gonorrhoeae* in blood, synovial fluid, or tissue.^2^ Presumptive diagnosis can be made in a patient with a clinical presentation consistent with disseminated gonorrhea and microbiologic evidence from the urogenital, rectal, or pharyngeal tract.^2^ ED treatment should include ceftriaxone one gram intravenously and azithromycin one gram orally, and the patient should be admitted for further management.^2^

**CPC-EM Capsule**

What do we already know about this clinical entity?

Although a relatively rare entity, cases of disseminated gonorrhea have become more prevalent with the recent increase in sexually transmitted infections in the United States.

What is the major impact of the image(s)?

Cutaneous manifestations of disseminated gonorrhea are varied but can present as hemorrhagic bullae.

How might this improve emergency medicine practice?

When faced with an abnormal skin lesion, the emergency physician should gather further history into the sexual practices of the patient.

## Figures and Tables

**Image:**
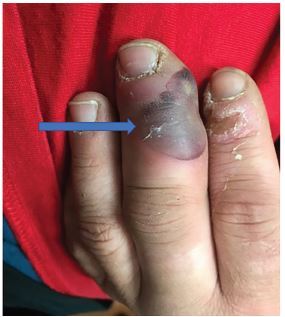
Photograph of the left third digit depicting edema, erythema, and a hemorrhagic bullae (arrow).
